# Discrete Typing Units of *Trypanosoma cruzi* Identified by Real-Time PCR in Peripheral Blood and Dejections of *Triatoma infestans* Used in Xenodiagnosis Descriptive Study

**DOI:** 10.3390/pathogens11070787

**Published:** 2022-07-12

**Authors:** Inés Zulantay, Gabriela Muñoz, Daniela Liempi, Tamara Rozas, María José Manneschi, Catalina Muñoz-San Martín, Carezza Botto-Mahan, Werner Apt, Gonzalo Cabrera

**Affiliations:** 1 Laboratorio de Parasitología Básico-Clínico, Programa de Biología Celular y Molecular, Facultad de Medicina, Instituto de Ciencias Biomédicas, Universidad de Chile, Santiago 8330015, Chile; gabriela.munoz@um.uchile.cl (G.M.); daniela.liempi@uach.cl (D.L.); tamara.rozas@ug.uchile.cl (T.R.); wapt@med.uchile.cl (W.A.); 2 Programa de Magister en Parasitología, Facultad de Medicina, Universidad de Chile, Santiago 8330015, Chile; 3 Instituto de Parasitología, Facultad de Medicina, Universidad Austral de Chile, Valdivia 5090000, Chile; 4 Laboratório de Biologia de Trypanosamatídeos, Instituto Oswaldo Cruz/Fundação Oswaldo Cruz, Rio de Janeiro 21040-900, Brazil; mariamanneschi@gmail.com; 5 Escuela de Medicina Veterinaria, Facultad de Ciencias Médicas, Universidad Bernardo O’Higgins, Santiago 8370993, Chile; catasanmartin@hotmail.com; 6 Departamento de Ciencias Ecológicas, Facultad de Ciencias, Universidad de Chile, Santiago 7800003, Chile; cbotto@uchile.cl; 7 Laboratorio de Biología Celular y Molecular, Programa de Biología Celular y Molecular, Instituto de Ciencias Biomédicas, Facultad de Medicina, Universidad de Chile, Santiago 8385403, Chile; gonzalocabrera@uchile.cl

**Keywords:** DTUs, *Trypanosoma cruzi*, real-time PCR, peripheral blood, xenodiagnosis, *Triatoma infestans*

## Abstract

Chagas disease (ChD) is a vector zoonosis native to the American continent caused by the protozoan parasite *Trypanosoma cruzi*; the biological vectors are multiple species of hematophagous insects of the family Triatominae. A relevant aspect in the host–parasite relationship is the identification of the various genotypes of *T. cruzi* called discrete typing units (DTU) that circulate in mammals and vectors. In Chile, it has been described that the DTUs TcI, TcII, TcV, and TcVI circulate in infected humans, vectors, and wild animals. Identifying DTUs has acquired clinical importance, since it has been suggested that different genotypes could cause distinct pathologies, circulate in different geographical areas, and present different sensitivities to trypanocidal drugs. In this study, circulating *T. cruzi* DTUs in peripheral blood and *Triatoma infestans* dejections used in xenodiagnosis (XD) were amplified by qPCR in 14 Chilean patients with chronic ChD from highly endemic areas. More positive samples were detected by XD compared to peripheral blood samples, and 64.28% of the cases were simple infections and 35.72% mixed, with a statistically significant difference in the frequency of TcV DTU. This study would suggest that *T. infestans* from Chile is more competent to amplify one DTU over others, probably due to a process of co-evolution.

## 1. Introduction

Chagas disease (ChD) is caused by *Trypanosoma cruzi* and is transmitted by blood-sucking insects of the subfamily Triatominae (Hemiptera: Reduviidae). It is considered the most relevant neglected disease in the American continent; the vast majority of those infected come from rural areas. ChD is present in 21 countries, from the southern United States to southern Argentina in South America, with the exception of the Caribbean islands, and it mainly affects Latin American countries [1]. In Chile, ChD extends from the Arica and Parinacota Region (18°30′ S) to the Libertador Bernardo O’Higgins Region (34°36′ S). It is estimated that 160,000 people are infected by *T. cruzi* in the country [2]. In 1999, Chile was declared free of vector intradomicile transmission by *T. infestans* [3], although populations of domestic triatomines continue occurring in the wild [4]. *T. infestans* is the vector of the domestic cycle and *Mepraia spinolai*, *M. gajardoi*, and *M. parapatrica* are of the wild cycle [5].

By international consensus, *T. cruzi* strains are currently divided into six monophyletic clades: TcI, TcII, TcIII, TcIV, TcV, and TcVI [6], plus a seventh DTU identified in bats called TcBat [7]. The concept of discrete typing unit (DTU) refers to a group of *T. cruzi* strains that are more similar to each other than to other strains and are identifiable by molecular markers [8]. DTUs can be considered reliable units of analysis for molecular epidemiology and experimental evolutionary studies. TcI is the DTU with the widest geographic distribution and the greatest internal genetic diversity, with transmission cycles that overlap between domestic and wild species [9,10]. TcII, TcV, and TcVI are predominantly found in the Southern Cone countries [11]. The identification of DTUs present and the diversity within them is important, since different genotypes may cause different pathologies and circulate in different locations and transmission cycles, affecting control, treatment, and vaccine development efforts [12].

The circulating genotypes in humans and vectors are diverse in Chile. According to Campos-Soto et al. [13], mixed infections of TcI, TcII, TcV, and TcVI DTUs have been detected in the wild triatomines *M. gajardoi* and *M. spinolai* from the north-central zone of the country. *M. gajardoi* fed by xenodiagnosis (XD) on *Octodon degus* rodents were more competent to maintain and transmit TcI and TcVI and less TcV [14]. TcI was detected in *T. infestans* collected within the Metropolitan Region in 100% of domestic vectors and in 93% of wild vectors, in both single and mixed infections. The wild populations had a higher number of mixed TcII, TcV, and TcVI infections [15]. In *O. degus*, droplets of *T. infestans* (domestic vector) feces after XD amplified TcI more than droplets of *M. spinolai* (wild vector) feces, probably because the *T. infestans* population in Chile are less adapted to wild cycles [16].

For some years, it has been supported that there is differential transmissibility of clonal genotypes by *T. infestans* under experimental infection conditions [17,18,19,20]. The combination of an XD test, cell culture, and various genotype typing strategies such as DNA hybridization, isoenzyme identification, and multilocus sequence typing, among others, has raised new questions regarding the transmissibility of *T. cruzi* [19,20]. Ortiz et al. [21] detected the DTUs of patients with ChD from the Province of Choapa in the semi-arid north of Chile by means of DNA hybridization, obtaining TcI, TcII, TcV, and TcVI. They obtained only TcV by XD and axenic culture.

The objectives of this descriptive study were to compare: (i) the parasite load in blood samples and dejections of XD, (ii) the DTUs of *T. cruzi* circulating in a group of individuals with chronic ChD, through real-time PCR (qPCR) in peripheral blood samples (qPCR-BS) and droplets of axenic *T. infestans* (qPCR-XD), and (iii) the frequency pattern of *T. cruzi* DTUs according to the type of biological sample analyzed.

## 2. Results

### 2.1. Parasite Load in Blood Samples and Dejections of XD

Most of the peripheral BS had a low *T. cruzi* parasite load, with an average of 17.91 ± 22.93 par. eq/mL and a median of 11.23 par. eq/mL. The lowest value of qPCR-BS was obtained in patient 13, with 0.72 par. eq/mL, and the highest in patient 9, with 90.4 par. Eq/mL. In all the cases with positive XD, it was possible to determine the parasite load of *T. cruzi* (Table 1). Patient 4 had the lowest parasite load, with 1.79 par. eq/mL, and patient 1 had the highest, with 140,650 par. eq/mL. The mean parasite load of qPCR-XD was 10.512 ± 37.47 par. eq/mL, with a median of 147.25 par. eq/mL. Patients 3 and 4 did not amplify for any of the DTUs analyzed; they presented parasite loads of 12.65 and 1.79 par. eq/mL, respectively. All trials were performed in duplicate. Statistically significant differences (U = 29, *p* < 0.05) were detected between the parasite loads of the two biological samples.

### 2.2. Genotyping in Blood Samples and Dejections of XD

*T. cruzi* was genotyped in dejections of XD in 12/14 patients (85.71%). In patients 2, 4, and 11, simple infections by DTUs Tcl, TcII, and TcV were detected, respectively. It was not possible to genotype *T. cruzi* in 9/14 peripheral BS (64.28%). In the five remaining cases (35.71%), four had a single infection, and one had a mixed infection (Table 2). More genotypes were detected in dejections of XD than in BS (X^2^ = 7.34, *p* < 0.01). Among the identified DTUs, *T. infestans* preferentially amplified TcV over other DTUs (X^2^ = 7.04, *p* < 0.01). The results of the external control genotyping with TcI, TcII, and TcV presented specific Tm within the expected range.

Single infections (with a unique DTU) were detected by qPCR-XD in seven patients (50%), while mixed DTUs were detected in five cases (35.7%). No DTU was detected in two patients (14.3%). In single infections, DTU TcII was detected in one case (7.14%) and TcV in six (42.85%). In mixed infections, TcI + TcV were detected in two patients (14.28%) and TcII + TcV in three patients (21.42%). No significant difference was found in the frequency of single and mixed infections (X^2^ = 2.29; *p* = 0.13).

TcV was the most significantly detected DTU by qPCR-XD (X^2^ = 14.03, *p* < 0.001) compared to TcI and TcII. In the BS, nine patients were negative (64.28%), one with TcI (7.14%), three with only TcV (21.42%), and one with Tcll + TcV (7.14%). Table 2 details the percentage of DTUs in XD and BS.

At least in one of the two XD samples it was not possible to genotype DTUs, but low parasite burden was observed. During the qPCR assays, some dissociation curves were generated that were not coincidental with the specific temperature for one of the three DTUs investigated, which can be attributed to the presence of other DTUs not analyzed in the samples or in specific amplification processes.

## 3. Discussion

Circulating genotypes could only be detected in 5/14 BS (35.71%). It is likely that the low parasite burden and intermittent release of trypomastigotes to the bloodstream, characteristic of chronic ChD infections [22], explain the low frequency of identification of *T. cruzi* DTUs. In this study, 50% of the cases had qPCR values below the mean and the median obtained for peripheral BS. Saavedra et al. [23] described that most peripheral BS of Chilean patients evaluated by qPCR had less than 10 par. eq/mL, in agreement with what was described by other authors for patients from Brazil, Argentina, and Colombia, with medians of 1.18, 1.95, and 2.31 par./eq/mL [24,25].

The most frequent DTU detected in BS was TcV (21%), followed by TcI (7%), and TcII + TcV (7%). In a larger sample with *T. cruzi* genotyping in BS of patients with chronic ChD, performed with hybridization probes of kinetoplastid DNA minicircles, the most frequent DTUs in decreasing order were TcV, TcI, TcII, and TcVI [26]. A simple infection was detected in nine patients (64.28%), and the remaining five cases had mixed infections by two DTUs (TcI + TcV and TcII + TcV). Although TcVI was not included in our study due to its low frequency in people with ChD in Chile, it has been previously described [27,28], so it could have been present in BS that could not be genotyped.

Taking into consideration the analytic performance of genotypes by PCR using TaqMan probes, Cura et al. [29] could not type a proportion of BS samples of chronic ChD patients with a low parasite burden. This observation may be closely related to the sensitivity of *T. cruzi* DTU typing methods obtained directly from peripheral BS. Unpublished data obtained in our laboratory, in at least two years of experimental work, indicated that it is not possible to genotype circulating populations of less than 10 par. eq/mL. Considering that a high percentage of individuals with chronic ChD present a parasite burden below this limit of detection [23], a large percentage of cases could not be genotyped under the conditions described in this paper. Therefore, it is necessary to carry out an analytical validation of the qPCR technique to determine the sensitivity and analytical specificity of the circulating DTUs of Chile.

More complete results were obtained with the qPCR-XD probe, since more positive cases were detected compared to the BS [30], amplifying more *T. cruzi* DTU TcV, similar to what has been described [21]. The same DTUs could be genotyped in XD samples and BS in cases 8, 9, and 10. However, in case 9, qPCR-XD detected TcII and TcV, and in BS, only TcV could be genotyped. The qPCR-XD technique showed a very wide range of parasite loads, from less than 1 par. eq/mL to more than 100,000 par. eq/mL.

Previous genotyping studies with hybridization probes in peripheral BS and XD-*T. infestans* described different frequencies; DTU TcI was the most frequent in peripheral BS and TcV in XD-*T. infestans* [31]. In Ortiz et al. [21], DTUs detected directly in the peripheral BS of patients with chronic ChD disease with hybridization probes included TcI, TcII, TcV, and TcVI, while only TcV was detected by XD and axenic culture. It has been suggested that these differences between humans and triatomines represent the association of the hosts with *T. cruzi* genotypes [31]. An alternative explanation of the preference of DTUs by *T. infestans* is that *T. infestans* reduces the composition of *T. cruzi* in axenic cultures and the transmission in the vector is assimilated to the generation of a clonal population [21]; although, in other experiments, a competitive trend between genotypes was not detected [20]. It has been observed that the parasite clones have adapted to vertebrates and invertebrates [31]. An alternative explanation is that the triatomines used in Chile for XD have acquired locally a biological filter that affects the specific amplification of original DTUs. The original colony of *T. infestans* used for XD comes from a parental sample of triatomines captured in the 1950s in various locations in the Coquimbo Region [16,32]. Genetic diversity may have been reduced as a founder effect in the capture process, in addition to the decades’ reproduction of *T*. *infestans* under the laboratory conditions. Genetic and morphological differences have been described between samples of wild, domestic, and laboratory colonies of triatomines [33,34,35,36]. Higher amplification of TcI followed by TcV and TcII was observed in vector transmissibility studies using colonies of *T. infestans* from Chile, Uruguay, and Brazil [17,18,20]. The selection of genotypes has also been observed in studies with cultures of *T. cruzi* [19].

It has been demonstrated that, apart from the ecological and epidemiological peculiarities, the genotypes present biological and clinical differences of transmissibility, virulence, and susceptibility to drugs [37,38,39,40,41]. Experimental studies have shown that vector transmission in *T. infestans* is not homogeneous among clonal genotypes [17,21]. The experimental infection of *T. infestans* nymphs with TcI, TcII, and TcV clonal lines found greater transmissibility with TcI, intermediate with TcV, and low with TcII [17]. Using the same clonal genotypes, but this time with mixed pairs (TcI + TcII, TcI + TcV, TcII + TcV), the behavior also differed between clones. The authors suggested an interaction between the genotypes involved in the mixture, with some cases of inhibition or reciprocal inhibition [18]. Da Silveira Pinto et al. [20], again with the same bi-clonal pairs, by detection with hybridization probes, reported a differential efficiency in transmission between genotypes, without a tendency for one genotype to eliminate another. In addition, to evaluate the infective capacity of clonal lines grown in vitro in Vero cell lines, they showed by phylogenetic distancing that the trypomastigote forms of TcI were more infective than TcII and TcV [39].

The interaction and competition of *T. cruzi* genotypes have also been described in vertebrates, specifically in infected BALB/c rodents with genotypes TcI, TcII, and TcV to evaluate the biological properties of *T. cruzi*. They observed that, in mixed infections, the genotypes did not behave as monoclonal infections but showed significant differences in phylogenetically distant mixtures [42]. Mice infected with DTU clones TcI, TcII, and TcIV differed in detection according to the diagnostic technique used in the study (blood culture, ELISA, PCR) [41]. *T. infestans* and *M. spinolai* used for XD on wild and naturally infected *Octodon degus* showed variable susceptibility to clonal lines of *T. cruzi*. The wild vector *M. spinolai* presented a greater capacity to host and reproduce different genotypes of local circulation compared to *T. infestans* [16]. These experimental studies should be extrapolated to epidemiological patterns with caution [39]. *T. cruzi* characterization techniques are multiple and varied and could have effects on the detection of clonal genotypes [18,20]. It has been shown that the population dynamics of different strains of the parasite were altered depending on the vector species, XD even distinguishing between wild and domestic triatomines [16,43,44]. The cause would be found in the vector, since this insect itself is genetically heterogeneous and interferes in its vectorial competence [17]; thus, the disadvantage of using axenic triatomines is that they could affect the final results of the experiment. In future studies, this should not be a reason not to use it, but to understand that the vector is an organism with its own co-evolutionary history with *T. cruzi* in the American continent. Another methodological explanation for the low number of DTUs in blood and the high frequency detected for one DTU compared to others could be due to the problems surrounding nucleic acid amplification techniques in PCR. It is possible that inhibitors present in the sample have affected the performance of PCR [45] or the presence of strains with different polymorphisms that were not detected by the primers used [27]. The non-specific amplifications reported could be explained by the circulation of another genotype, probably TcVI or a variant of TcI. The design of the primers used consisted of two Tcl strains: House 510 C8C3 for TcIa and Dm28c for Tclb, based on the SL-IR polymorphism [27]. Genotypes TcIa and TcIe circulate in Chile, and the latter is associated with the wild cycle of *M. gajardoi* and *M. spinolai* [46]; thus, the addition of more specific qPCR primers would have increased the detection of mixed infections. It is important to consider that after 30, 60, or 90 days, *T. infestans* has the capacity to exponentially amplify *T. cruzi* present in the initial inoculum, increasing the concentration of extracted DNA and favoring the genotyping. Finally, it should be considered that the integrity of the parasitic DNA present in these samples may have been altered due to the different variables, depending on the storage and treatment of the sample.

The different species of triatomine vectors have a geographic distribution in niches and hosts to which they have adapted [47,48], which in turn are inserted in a complex network of host–vector–parasite transmission cycles where there are recurrent genera of reservoir mammals and *T. cruzi* lineages. In Chile, known triatomine vectors and wild and domestic mammals present different frequencies of infection with *T. cruzi*, with spatial and temporal variations [49,50], thus maintaining the parasite [13,51,52]. In the rodent *O. degus*, a very abundant species in the semiarid Mediterranean ecosystem, DTU TcI of *T. cruzi* has been reported with greater occurrence; although, in other wild rodents and *Carpra hircus* from the Coquimbo Region, there is no preference or specificity of DTUs for any species [52].

In synthesis, it has been observed that various animals and triatomines that have been genotyped do not share the same frequencies of DTUs. According to the latest qPCR detections in patients with chronic ChD in Chile, the circulating DTUs with the highest frequencies are TcI, TcII, TcV, and TcVI [27,28]. Then, why is there a significant higher frequency of TcV DTU obtained by XD of *T. infestans*? Could DTUs be interacting competitively as previously suggested [31]? Or would TcV be more prevalent in the geographic area where this study was performed?

It seems that axenic triatomine experiments are not a reflection of the patterns of DTUs of the transmission cycles of hosts in the semi-arid ecosystem of Chile. Those circulating DTUs that have co-evolved in *T. infestans* would be detected more frequently. Co-evolution is frequent in parasitic interactions, and there are certainly cases in which the host and parasite have a reciprocal evolution [53]. As it has already been observed in samples from the same case, the absence of TcV in blood and its occurrence in axenic vectors would indicate that this DTU was already present in the patient, in a very low parasitic load, and that it was later amplified probably by a reciprocal adaptation, which allows us to propose future studies. *T. infestans* is a species of recent appearance, widely distributed in several geographical areas, that inhabits seven countries in South America, apparently due to human migrations of the last 100 years [54]. Genetic research has postulated that there are two major evolutionary lineages, an Andean and a non-Andean lineage, plus a third intermediate lineage [55]. There are two hypotheses about the origin: the valleys of central Bolivia [55,56] and the subtropical low zone of the Gran Chaco [57]. TcV and TcVI are frequent genotypes in the southern zone of South America that circulate in the domestic transmission cycle, for example, in humans and dogs, they are rarely found in wild cycles [58], and they would have emerged from at least two hybridization events between TcII and TcIII [59,60]. Their origin is probably in the Chaco area and adjacent Andean valleys [60]. It is estimated that these two genotypes arose less than 60,000 years ago; however, they were fixed in the domestic cycle by a “bottleneck” type genetic drift event from those congregated around the domicile and peridomicile of pre-Hispanic populations [61]. According to Usinger et al. [62], the approach of *T. infestans* could have occurred in approximately 5000 B.C., as consequence of the domestication of wild guinea pigs (similar to *Cavia tschudii*) by the Andean tribes of Bolivia, Perú, and Chile. The hypothesis of an anthropogenic intervention seems plausible. However, archaeological evidence and the comparison of phylogenies are lacking. Therefore, caution should be suggested until these studies are carried out.

In conclusion, different profiles of *T. cruzi* DTUs are obtained from two biological samples taken in parallel in chronic ChD patients. TcV is the *T. cruzi* DTU more frequently isolated in samples of triatomine dejection obtained by XD. This result could be a consequence of an initial inoculum with a higher parasite load of TcV derived from the infected patients. The BS represents the diversity of *T. cruzi* DTUs that circulate in patients with ChD from the semi-arid ecosystem of Chile. On the contrary, when using dejections of patients XD-*T. infestans*, the human DTUs more related to the triatomines would be amplified. The causes of this higher probability of amplification are unknown, although it could not be discarded that it is linked to a process of co-evolution.

## 4. Materials and Methods

### 4.1. Study Population

14 patients with chronic ChD, seven men and seven women, confirmed by conventional serology (ELISA and IFI IgG) according to the methodology described [63], studied in controls for this parasitosis in urban and rural health centers of the Province of Choapa (Illapel and Salamanca) and Limarí (Combarbalá), Chile, were included in this study. The women’s ages ranged from 20 to 53 years old, with an average of 46.9 years old. Men’s ages ranged from 32 to 76 years old, with an average of 57.4 years old. The patients agreed to take part in the study under Informed Consent (FONDECYT Project 1100768), approved by the Ethics Committee of the Faculty of Medicine, University of Chile (Resolution 046-2009). The inclusion criteria that limited the number of cases in the study group considered: 1. people without previous trypanocidal treatment 2. coming from the same endemic area (rural or urban sector) 3. availability of genomic DNA from peripheral BS 4. availability of genomic DNA obtained from dejection samples in XD after 30, 60, and 90 days of incubation and a pool prepared with these samples 4. both samples (BS and XD) obtained in parallel (same day and time of control) 5. presence of *T. cruzi* detected in peripheral blood by means of conventional PCR prior to the study by observing the specific band of *T. cruzi* of 330 bp detected with the 121–122 kinetoplastid starters and 6. positive microscopic study (observation of motile *trypomastigote* forms) from droppings obtained from xenodiagnosis at 30–60 and 90 days of incubation. Patient data and XD results are described in Table 3.

### 4.2. Biological Samples of Xenodiagnosis

XD was applied to the studied patients according to the technique described by Schenone [64]; it consisted of two cylindrical wooden boxes covered by a piece of gauze, each with seven nymphs of the third or fourth instar of *T. infestans* free from infection. They were subjected to a fast for a period of 3–4 weeks. The insects were fed for 20 to 30 min on the dorsal side of each patient arm then the nymphs were incubated at 27 °C and 75% humidity in an incubation chamber. After 30, 60, and 90 days, triatomine fecal samples were obtained by abdominal compression in a biosecurity chamber; they were analyzed microscopically, searching for motile trypomastigote forms of *T. cruzi*. Both sampling boxes were positive in some cases. Afterwards, a pool of the dejections analyzed at 30, 60, and 90 days was prepared, diluted in 500 μL PBS pH 7.2 The pool was incubated for 15 min at 98 °C and centrifuged for 3 min at 3500 rpm. The supernatant of 200 µL was stored at −20 °C until its use (while XD is very useful for verifying the presence of viable *T. cruzi*, we are not currently applying this diagnostic tool) [23,30,65].

### 4.3. DNA Extraction of Dejections Obtained from Xenodiagnosis (qPCR-XD)

Before DNA extraction from the dejection, 20 μg of DNA of human blood negative for *T. cruzi* was added as an exogenous internal control. DNA purification was performed using an initial volume of 100 μL, with the genome DNA extraction kit Favor Prep Blood Mini kit (Favorgen, Biotech Corporation), modified by the omission of cell lysis with K proteinase. The eluted was preserved at −20 °C until qPCR-XD was performed [23,65].

### 4.4. Obtention and Purification of DNA from Blood Samples (BS) for qPCR (qPCR-BS)

Peripheral BS was obtained by venipuncture and was stored in Venoject tubes with guanidine HCl-EDTA in 1:1 volume (2 mL blood in 2 mL of preservant anticoagulant solution). The samples were incubated at 98 °C for 15 min to favor the breaking of *T. cruzi* minicircles and were conserved at 4 °C until DNA extraction. DNA purification was performed with an initial volume of 200 µL, according to the instructions of the kit QIA amp DNA Blood Mini Kit (Qiagen). The eluted was conserved at −20 °C until qPCR-BS was carried out [27,28,30].

### 4.5. PCR in Real Time (qPCR)

To obtain DNA and generate the standard curve to quantify the parasite charge of *T. cruzi* by PCR, a stock of trypomastigote forms of *T. cruzi* strain DM28c e Y obtained from axenic culture were used. Considering that one parasite cell harbors approximately 200 fg of DNA, the standard curve was performed with a 10-fold serial dilution of DNA extracted from BS of healthy donors spiked with 10^6^ par. eq/mL. Given the variability in the number of copies of the nuclear satellite DNA previously described, the curve was made with equal quantities (20 ng of each one/200 μL blood) of the clonal reference strains Dm28c (TcI highest number of copies) and Y (TcII lowest number of copies) to reduce the differences in the detection limits [23,24,26,30]. 

### 4.6. Trypanosoma cruzi Quantification

The parasite load in the study samples was quantified by qPCR assays with the SYBR^®^ Green Mx3000PTM Stratagene detection system (Agilent Technologies, Santa Clara, CA, USA), using the DNA primers nuclear cruzi 1 (5′-ASTCGGCTGATCGTTTTCGA-3′) and cruzi 2 (5′-AATTCCTCCAAGCAGCGGATA-3′) at a concentration of 0.3 µM. The final reaction volume was 20 µL, containing 2 µL of template DNA, 1X Brilliant III Ultra-Fast SYBR^®^ Green qPCR Master Mix (Agilent Technologies), 0.3 µM of primers, and reference dye (ROX). The conditions of the reaction included a pre-incubation for 1 s at 25 °C and 3 min at 95 °C, followed by 40 cycles at 95 °C for 5 s and 60 °C for 20 s. Nuclease-free water and seronegative human DNA were used as negative controls. Reference DTUs TcI (Dm28c), TcII Y, and TcV (92.80) were used as positive controls. A negative result was considered (no detection of *T. cruzi*) when the value of the fluorescence emission was not quantifiable. The positive results were informed as par. eq/mL [23,24,26,27,65].

### 4.7. Genotyping by Real-Time PCR

The genotyping process was conducted by applying the SYBR^®^ methodology to identify DTUs present in *T. cruzi* in each of the samples whose parasitemia was previously demonstrated qualitatively by conventional PCR and quantitatively by qPCR. The DTUs of TcI, TcII, and TcV were investigated, which are the most frequent genotypes in Chile. The primers designed for TcI (Gen SL-IR) Fw 5′-GCTTTGTGTTCTCGCACTCCA-3′ (0.4 µM) and TcI (Gen SL-IR) Rv 5′-CGATCAGCGCCACAGAAAGT-3′ (0.4 µM), for TcII (Gen COII) Fw 5′-GGATTACATCTACGGCTGACACC-3′ (0.2 µM) and TcII (Gen COII) Rv 5′-CGAGAGTGATTATTTGGTGGGAGATA-3′ (0.2 µM), for TcV (Gen ND1) Fw 5′-AGTTATTCATTAATCTTATG-3′ (0.5 µM) and TcV (Gen ND1) Rv 5′-CCATCTGTGATAGGTGTTAATATTCC-3′ (0.5 µM) were used. The samples were processed in duplicate, and each assay included a non-template control (nuclease-free water), a positive control (20 fg/μL of reference genomic DNA corresponding to the assay), and a negative control (20 fg/μL of reference genome not corresponding to the DTU of the assay and DNA of seronegative human blood). The reference strains used were Dm28c (TcI), Y (TcII), and 92.80 (TcV) [26,27,28].

### 4.8. DTU Identification of T. cruzi

According to Muñoz et al. [27], the different DTUs were identified by different primers, and the correct amplification was confirmed by analyzing dissociation melting curves. The specific Tm (melting temperature) for each expanded segment in each DTU was 86.8 °C (TcI), 77.8 °C (TcII), and 77.7 °C (TcV). An error of 0.25 in the peak of association curves was accepted. A variation of up to 0.25 °C can occur between trials; however, the primers are highly specific. 

### 4.9. Validation of the DTUs Genotyping Protocol

Dm28c (TcI), Y (TcII), and 92.80 (TcV) strains were also genotyped using a Rotor-Gene^®^ Q (QIAGEN, Hilden, Germany) thermocycler, in the Ecology Laboratory of the Faculty of Veterinary and Livestock Sciences of the University of Chile, using the same primers, concentrations, and controls as previously described in [27,28,65]. 

### 4.10. Statistical Analysis

To test for differences in the composition and load of the infectant DTUs between the peripheral blood of patients with ChD and dejections of triatomine vectors, Chi-square tests or the Mann-Whitney U test were used, respectively.

## Figures and Tables

**Table 1:** Results of qPCR amplification in peripheral blood samples and dejections of *Triatoma infestans* used in xenodiagnosis by patient, gender, age, *Trypanosoma cruzi* quantification (par. eq/mL), and DTU.

Patient	Sex	Age	Xenodiagnosis		Peripheral Blood	
			par. eq/mL	DTU	par. eq/mL	DTU
1	M	32	140,650	TcII	4.79	-
2	M	58	101.5	TcV	6.88	-
3	F	53	12.65	-	22.4	TcV
4	F	53	1.79	-	31.1	TcI
5	F	33	221	TcV	17.6	-
6	F	63	51.5	TcV	26.75	-
7	M	54	134	TcI-TcV	4.76	-
8	M	68	51	TcV	13	TcV
9	M	76	1388.50	TcII-TcV	90.4	TcV
10	F	20	653.5	TcII-TcV	9.46	TcII-TcV
11	F	53	160.5	TcV	4.24	-
12	M	56	3277	TcI-TcV	1.55	-
13	M	58	9.37	TcII-TcV	0.72	-
14	F	53	486.5	TcV	17.2	-

**Table 2:** Percentage of single and mixed infections in samples of XD and peripheral blood by DTU of *Trypanosoma cruzi* genotyped by real-time PCR.

*Trypanosoma cruzi* DTU	Xenodiagnosis %	Peripheral Blood Samples %
TcI	0	7.14
TcII	7.14	0
TcV	42.85	21.42
TcI + TcV	14.28	0
TcII + TcV	21.42	7.14
Undetected DTU	14.28	64.28%

**Table 3:** Patients with chronic Chagas disease and positive xenodiagnosis at 30–60 and/or 90 days, from the Coquimbo Region, Chile.

Patient	Sex	Age	Xenodiagnosis			Location
			30 Days	60 Days	90 Days	
1	M	32	(−)	(+)	ND	Illapel
2	M	58	(−)	(+)	(−)	Combarbalá
3	F	53	(−)	(−)	(+)	Combarbalá
4	F	53	(+)	(−)	(+)	Salamanca
5	F	33	(−)	(+)	(+)	Combarbalá
6	F	63	(−)	(+)	(+)	Combarbalá
7	M	54	(−)	2 (+)	2 (+)	Combarbalá
8	M	68	(−)	2 (+)	2 (+)	Combarbalá
9	M	76	2 (+)	2 (+)	2 (+)	Combarbalá
10	F	20	(−)	(+)	(+)	Salamanca
11	F	53	(−)	2 (+)	2 (+)	Salamanca
12	M	56	(+)	2 (+)	2 (+)	Illapel
13	M	58	(+)	(+)	(+)	Illapel
14	F	53	2 (+)	(+)	(+)	Salamanca

Xenodiagnosis positivity: 1 sampling box (+), two sampling boxes: 2 (+) and without microscopic detection of trypomastigote forms of *T. cruzi*: (−).
